# Calcium-Sensing Receptor and Regulation of WNK Kinases in the Kidney

**DOI:** 10.3390/cells9071644

**Published:** 2020-07-09

**Authors:** Daria S. Ostroverkhova, Junda Hu, Vadim V. Tarasov, Tatiana I. Melnikova, Yuri B. Porozov, Kerim Mutig

**Affiliations:** 1 Institute for Translational Medicine and Biotechnology, I.M. Sechenov First Moscow State Medical University (Sechenov University), 119991 Moscow, Russia; daria.ostroverkhova@gmail.com (D.S.O.); tarasov-v-v@mail.ru (V.V.T.); melnicov12@mail.ru (T.I.M.); 2 Department of Bioengineering, M.V. Lomonosov Moscow State University, 119991 Moscow, Russia; 3 Department of Functional Anatomy, Charité-Universitätsmedizin Berlin, 10115 Berlin, Germany; junda.hu@charite.de; 4 Department of Food Biotechnology and Engineering, ITMO University, 197101 Saint-Petersburg, Russia; 5 Department of Computational Biology, Sirius University of Science and Technology, 354340 Sochi, Russia

**Keywords:** with-no-lysine kinases, calcium-sensing receptor, distal nephron, NKCC2, NCC

## Abstract

The kidney is essential for systemic calcium homeostasis. Urinary calcium excretion can be viewed as an integrative renal response to endocrine and local stimuli. The extracellular calcium-sensing receptor (CaSR) elicits a number of adaptive reactions to increased plasma Ca^2+^ levels including the control of parathyroid hormone release and regulation of the renal calcium handling. Calcium reabsorption in the distal nephron of the kidney is functionally coupled to sodium transport. Apart from Ca^2+^ transport systems, CaSR signaling affects relevant distal Na^+^-(K^+^)-2Cl^−^ cotransporters, NKCC2 and NCC. NKCC2 and NCC are activated by a kinase cascade comprising with-no-lysine [K] kinases (WNKs) and two homologous Ste20-related kinases, SPAK and OSR1. Gain-of-function mutations within the WNK-SPAK/OSR1-NKCC2/NCC pathway lead to renal salt retention and hypertension, whereas loss-of-function mutations have been associated with salt-losing tubulopathies such as Bartter or Gitelman syndromes. A Bartter-like syndrome has been also described in patients carrying gain-of-function mutations in the CaSR gene. Recent work suggested that CaSR signals via the WNK-SPAK/OSR1 cascade to modulate salt reabsorption along the distal nephron. The review presented here summarizes the latest progress in understanding of functional interactions between CaSR and WNKs and their potential impact on the renal salt handling and blood pressure.

## 1. Introduction

Calcium homeostasis is critical to the intact cardiac rhythm and neuronal functions. Serum calcium levels are maintained within a narrow range of 2.2–2.6 mmol/L by orchestrated functions of many organs. The kidneys are essentially involved in this process via adjusting the calcium excretion to the needs of the body. To fulfill this task, renal transporting epithelia are equipped with receptors to the parathyroid hormone (PTH), calcitriol and calcitonin, enabling adaptive responses to systemic shifts of calcium homeostasis [1,2,3,4]. These endocrine mechanisms are complemented by the ability of kidney epithelia to directly sense serum calcium via the calcium-sensing receptor (CaSR) [5]. CaSR belongs to the family of G-protein coupled receptors. Upon binding Ca^2+^, CaSR activates the G proteins G_q/11_, G_i_, and G_12/13_, which stimulate phospholipase C (PLC)-dependent inositol 1,4,5-trisphosphate biosynthesis with subsequent Ca^2+^ release from intracellular stores (for review, see [6,7]). Intracellular Ca^2+^ [Ca^2+^]_i_ is an important second messenger affecting multiple Ca^2+^-sensitive enzymes to govern vital cell biological events such as gene transcription or vesicular transport [8]. Via these mechanisms, CaSR modulates expression, surface abundance, and phosphorylation of transport proteins mediating calcium reabsorption in kidney epithelia [9,10,11]. Since calcium handling in the distal nephron is functionally coupled to sodium transport, CaSR signaling has been also implicated in the volume homeostasis and blood pressure control [12,13,14,15]. The distal nephron comprises the thick ascending limb (TAL), the distal convoluted tubule (DCT), and the connecting tubule (CNT). The ensuing cortical collecting duct (CD) is functionally associated with the distal nephron, although it is derived from the ureteral bud like the other CD portions and does not belong to the nephron from the anatomical point of view [16,17,18]. There is growing evidence for CaSR-dependent regulation of the Na^+^-K^+^-2Cl^−^ cotransporter (NKCC2) in TAL and the Na^+^-Cl^−^ cotransporter (NCC) in DCT [12,13] (for review, see [19]). NKCC2 is critical to the urinary concentration and volume homeostasis [20,21]. NCC plays the key role in the fine tuning of urinary NaCl excretion and is further involved in the renal potassium handling [22,23] (for review, [24]). The extent of NaCl reabsorption in DCT determines the luminal Na^+^ load of the downstream CNT and CD, thus being a rate-limiting step for the Na^+^-reabsorption via the epithelial sodium channel (ENaC), which is electrogenically coupled to luminal K^+^-secretion via the renal outer medullary channel (ROMK, Kir1.1) in these segments [23] (for review, [25]). Loss-of-function mutations in genes encoding for NKCC2 or NCC cause hypokalemic salt-losing tubulopathies known as Bartter and Gitelman syndromes, respectively [26,27]. Similarly, gain-of-function mutations in the CaSR gene induce salt wasting due to suppression of NKCC2 activity [12,28]. Patients with Bartter syndrome including its CaSR-dependent form typically show activated renin-angiotensin-aldosterone system (RAAS) without concomitant pressor response, which can be explained by reduced angiotensin II (AngII) receptor sensitivity due to the chronic increase in plasma AngII levels. Altered prostadlandin metabolism may contribute to the impaired vascular reactivity in such patients as well [29]. Alternatively, inhibition of renal CaSR signaling has been shown to stimulate NKCC2 [30]. Excessive salt reabsorption in the distal nephron mediated by NKCC2 or NCC has been increasingly recognized as a relevant pathogenetic factor of salt-sensitive hypertension (for review, [31,32]). The two transporters belong to the family of electroneutral cation-coupled chloride cotransporters and share the posttranslational regulation by phosphorylation or dephosphorylation of conserved N-terminal threonine or serine residues (for review [33]). Their activating phosphorylation is provided by a kinase cascade comprising chloride-sensitive with-no-lysine [K] kinases (WNK) and two downstream kinases with high degree of homology, the Ste20/SPS1-related proline-alanine-rich kinase (SPAK) and the oxidative stress responsive kinase 1 (OSR1) [33]. Gain-of-function mutations in genes encoding for WNK1 or WNK4 cause Familial Hyperkalemic Hypertension (FHHt), also known as pseudohypoaldosteronism type 2 or Gordon’s syndrome [34]. Similarly, impaired ubiquitination and degradation of WNKs due to mutations in genes encoding for the kelch-like 3 (KLHL3) or cullin 3 have been associated with FHHt [35]. Enhanced phosphorylation of NCC by the activated WNK-SPAK kinase pathway has been recognized as the main pathogenetic mechanism of FHHt leading to renal salt retention, volume expansion, and hypertension [33,34]. Apart from the rare monogenetic hypertensive syndromes, WNK-SPAK-NCC signaling has been implicated in pathophysiology of salt-sensitive hypertension forms driven by RAAS hyperactivity or enhanced sympathetic tone [36,37]. Dephosphorylation of NKCC2 and NCC is mediated by calcineurin, which is a Ca^2+^/calmodulin (CaM)-dependent serine/threonine phosphatase [38,39,40]. Calcineurin suppresses the WNK-SPAK/OSR1 activity by promoting their degradation, which reduces the abundance of phosphorylated NKCC2 and NCC [39,40,41]. Therefore, functional interactions between Cl^−^-dependent and Ca^2+^-dependent phosphoenzymes determine the extent of salt reabsorption in TAL and DCT. Systemic shifts of extracellular calcium [Ca^2+^] levels may affect [Ca^2+^]_i_ in cells of the distal nephron via CaSR activity, thus modulating the renal salt handling. In this context, early epidemiologic studies demonstrated increased incidence of hypertension in individuals with restricted dietary calcium intake (for review, [42]). Moreover, individuals suffering from salt-sensitive hypertension appear to be particularly susceptible to reduced dietary calcium intake [43,44,45]. The purpose of this review work is to summarize the available information on the role of CaSR and Ca^2+^-signaling in the WNK-dependent regulation of distal salt reabsorption.

## 2. Renal Distribution of CaSR

**Figure 1:**
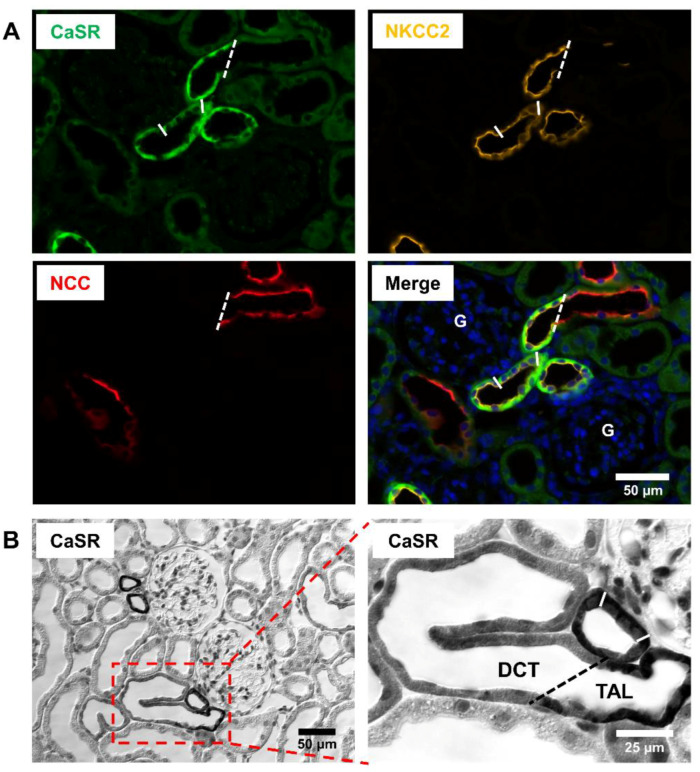
Calcium-sensing receptor distribution in the thick ascending limb (TAL) and distal convoluted tubule (DCT) of the rat kidney. (**A**) Representative fluorescence microscopic images demonstrating triple-labeling of a wild-type rat kidney section for the calcium-sensing receptor (CaSR; green signal), Na^+^-K^+^-2Cl^−^ cotransporter (NKCC2; yellow signal), and Na^+^-Cl^−^ cotransporter (NCC; red signal); nuclei are counterstained with DAPI (blue signal). A strong basolateral CaSR signal was detected in TAL including the macula densa cells (flanked by lines), as identified by concomitant apical NKCC2 signal. In contrast, CaSR signal intensity was low in the ensuing DCT identified by concomitant luminal NCC presence; the TAL/DCT transition is labeled by dashed line. (**B**) Representative differential interference contrasts microscopic image showing immunohistochemical labeling of a rat kidney sections for CaSR (left side) and a magnified insert (right side); macula densa cells are flanked by lines, TAL/DCT transition is labeled by dashed line. Note strong basolateral CaSR signal in TAL, as compared to weak, albeit significant labeling of the ensuing DCT. Scale bars account for 50 or 25 µm, as indicated in the respective images.

**Figure 2:**
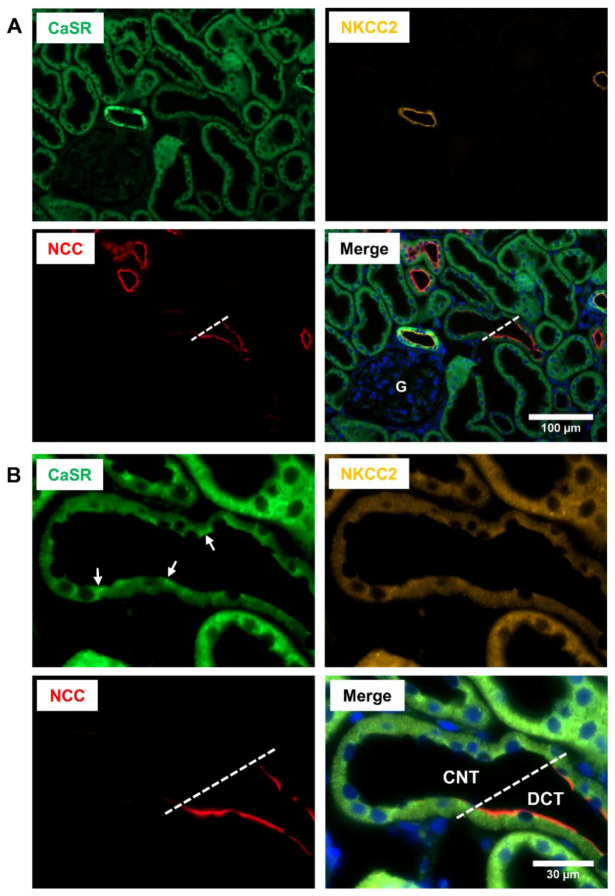
Receptor distribution in the distal convoluted tubule (DCT) and connecting tubule (CNT) of the rat kidney. (**A**) Representative fluorescence microscopic images demonstrating triple-labeling of a wild-type rat kidney section for the calcium-sensing receptor (CaSR; green signal), Na^+^-K^+^-2Cl^−^ cotransporter (NKCC2; yellow signal), and Na^+^-Cl^−^ cotransporter (NCC; red signal); nuclei are counterstained with DAPI (blue signal). A strong basolateral CaSR signal was detected in TAL identified by concomitant apical NKCC2 signal. The ensuing NCC-positive DCT and NKCC2/NCC-negative CNT showed moderate CaSR immunoreactivity partially at their luminal sides (arrows); the DCT/CNT transition is labeled by dashed line. (**B**) Magnification of the DCT/CNT transition region shown in (**A**). Scale bars account for 100 µm or 30 µm, as indicated in the respective images.

Since the cloning of mammalian CaSR, numerous efforts have been undertaken to determine its renal distribution, but the obtained results remain in part controversial. Initial analysis of CaSR expression sites in the rat kidney revealed presence of the CaSR mRNA in the glomerulus and along the entire nephron [2,46]. An alternative study reported the CaSR expression in the distal nephron and cortical CD but failed to detect it in the glomerulus or proximal tubule (PT) [47]. Therefore, despite controversy with respect to the proximal nephron part, the early studies unequivocally detected CaSR mRNA in the distal nephron segments using different techniques such as PCR from isolated nephron segments, in situ hybridization or northern blot [2,46,47]. At the protein level, an early work demonstrated significant CaSR abundance in the medullary and cortical TAL including the macula densa (MD) cells, as well as in DCT [48]. More recent well-controlled comparative analysis of CaSR distribution in the mouse, rat, and human kidneys verified its substantial abundance in TAL and DCT of all studied species using several antibodies recognizing different CaSR regions [5]. Controversial results have been obtained for cortical and medullary CD portions. An early study localized CaSR to a subset of type A intercalated cells (A-IC) [48], whereas more heterogeneous CaSR distribution in distinct CD cell types was described recently [5]. CaSR immunoreactivity in glomeruli and PT was low to absent, depending on the antibody and detection method [5,48]. At the cellular level, basolateral CaSR localization has been consistently documented in cortical and medullary TAL [5,48]. Depending on the study, basolateral or apical CaSR immunoreactivity was reported in DCT, and CD cells showed heterogeneous patterns as well [5,48]. For better comparison of CaSR distribution in distinct segments of the distal nephron, we performed a localization study in the rat kidney and documented transition portions between the distal nephron segments. To this end, we applied the anti-CaSR antibody from Abcam (ab19347) for immunofluorescence or immunohistochemistry [49]. We preferred this antibody because its specificity was extensively verified in previous work [5]. Our unpublished observations confirm substantial basolateral presence of CaSR in TAL including the MD cells (Figure 1). The ensuing DCT and CNT cells exhibit weaker CaSR signal, partially at the luminal side (Figure 1 and Figure 2). The available information on renal CaSR distribution has been also summarized and extensively discussed in a recent review work [7].

## 3. CaSR Function in TAL

TAL cells exhibit the most prominent CaSR abundance in the kidney. Cortical TAL is the site of paracellular reabsorption of Ca^2+^ and Mg^2+^ cations via claudin 16 (CLDN16) and CLDN19, which form heterodimeric paracellular divalent cation channels (for review [50]). The paracellular transport of cations is driven by the lumen-positive voltage generated by NKCC2 activity and luminal K^+^-recycling via the Kir1.1 potassium channel (rat outer medullary channel; ROMK) (for review, [20]). CaSR modulates both the transcellular and the paracellular transport capacity of TAL.

### 3.1. CaSR Inhibits the Transcellular NaCl Reabsorption

Effects of CaSR activation can be mediated by several interdependent signaling mechanisms including increased biosynthesis of eicosanoids, release of Ca^2+^ from intracellular stores, and decrease of intracellular cAMP concentration (for review [6,7]).

Eicosanoids have major impact on the transport function of TAL [51]. Early work showed that increases in [Ca^2+^]_o_ stimulate biosynthesis of cytochrome P450 and phospholipase A2-derived eicosanoids, which suppress the ROMK function [52,53]. Basolateral K^+^ transport in TAL is also sensitive to [Ca^2+^]_o_ and CaSR activity [54,55,56]. CaSR has been shown to decrease the surface expression and current activity of Kir4.1, which is one of the basolateral potassium efflux pathways in TAL cells (for review, [57]). Inhibition Kir4.1 or other basolateral K^+^-channels may secondarily affect the Cl^−^ efflux and modulate the transcellular NaCl transport in TAL cells, as has been previously described for DCT [23]. Therefore, reduction of the ROMK-mediated luminal K^+^ recycling or the basolateral K^+^ exit may underlie the CaSR-induced suppression of NKCC2-mediated NaCl reabsorption along the TAL [19]. Furthermore, CaSR-induced Ca^2+^ release from intracellular stores is expected to activate calcineurin. Calcineurin promotes cyclooxygenase 2 (COX-2) expression in TAL cells, which increases the bioavailability of COX-2-derived prostanoids including the prostaglandin E2 (PGE2) [58]. PGE2 interferes with transport activities of NKCC2 and NCC, thereby exerting significant inhibitory effects on the salt reabsorption along the distal nephron [59,60]. Little information is available on effects of eicosanoids on the WNK-SPAK/OSR1 activity. Suppression of renal COX-2 by calcineurin inhibitors (CNI) was associated with markedly increased kinase activity of WNK-SPAK/OSR1, hyperphosphorylation of NKCC2, and NCC and distal salt retention in rodents [40]. However, the role of COX-2-derived prostanoids is unclear in this setting, since CNI may affect the WNK-SPAK/OSR1 pathway via multiple other mechanisms both at cellular and systemic levels [38,39,61].

**Figure 3:**
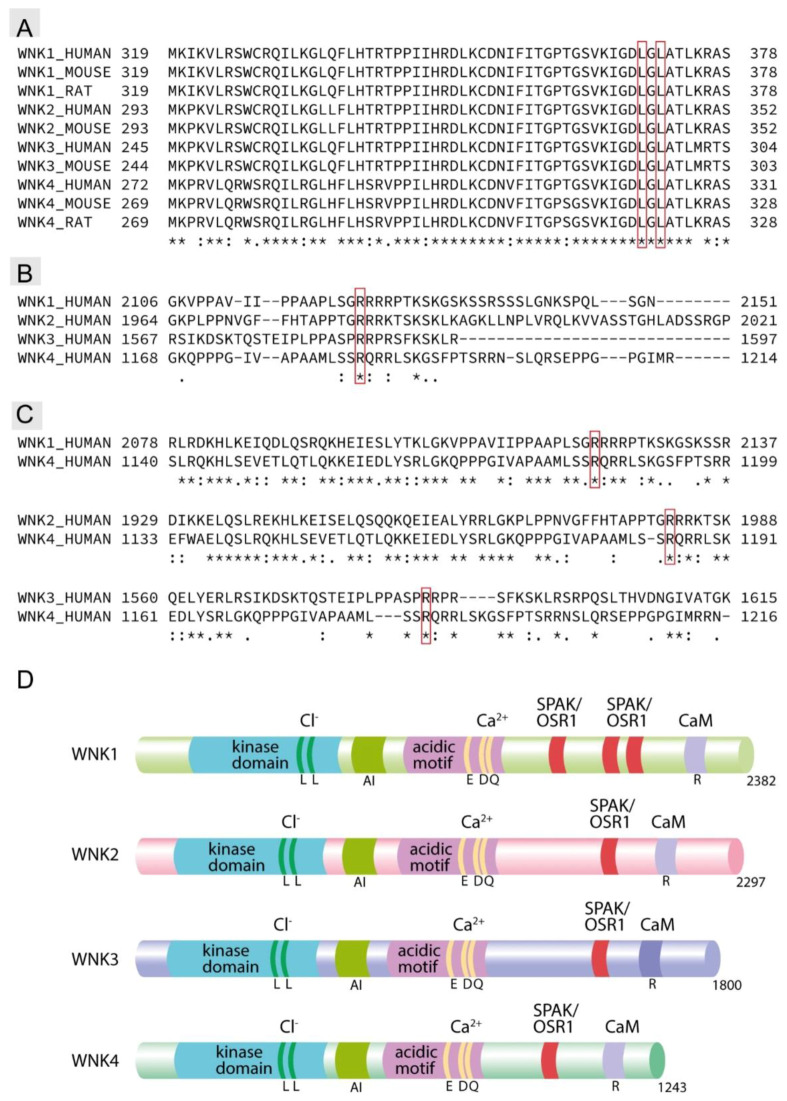
Multiple and local sequence alignment of with-no-lysine [K] (WNK) isoform. (**A**) Cross-species multiple sequence alignment (MSA) of all mammalian WNKs isoform. Lysine residues predicted to be relevant for chloride sensing are included into the red box (**B**) MSA of all human WNKs isoforms. Arginine residues predicted to be involved in calmodulin binding are included into the red box (**C**) Local sequence alignment of each WNK isoform against WNK4 showing a high degree of conservation of the putative calmodulin-binding motif between WNK1, WNK2, and WNK4, whereas WNK3 showed a lower degree of the motif conservation. Conserved arginine residues predicted to be involved in calmodulin binding are included into the red box. (*)—Fully conserved amino-acid residues, (:)—conservation via residues with highly similar properties, (.)—conservation via residues of lower similarity. (**D**) Schematic drawing illustrating putative Ca^2+^ binding sites within the acidic domains and C-terminal calmodulin (CaM) docking motifs across the human WNK isoforms. The conserved amino acid residues, which may mediate interactions with Ca^2+^ (E, D, and Q) or CaM (R) are specified. Kinase domains with conserved lysine residues mediating the chloride-sensitivity, auto-inhibitory domains (AI), as well as SPAK/OSR1-binding motifs are shown as well.

Nevertheless, CaSR signaling clearly interferes with posttranslational phosphoregulation of NKCC2 [30]. Kidney-specific CaSR deletion markedly increased levels of activating NKCC2 phosphorylation at its N-terminal threonine residues targeted by SPAK/OSR1, thus implicating the WNK-SPAK/OSR1 pathway in the CaSR signaling [30]. Notably, increased NKCC2 activity in kidney-specific CaSR-knockout mice was associated with the renal inability to excrete excessive dietary calcium load. This effect was independent on PTH, thus stressing the relevance of local CaSR signaling in TAL for the global calcium homeostasis [30]. Molecular pathways connecting CaSR to WNK-SPAK/OSR1 are not entirely clear but several possibilities can be taken into consideration. Since intracellular Cl^−^ [Cl^−^]_i_ exerts inhibitory effects on the catalytic WNK activity, CaSR-induced decrease of basolateral K^+^ efflux may lead to [Cl^−^]_i_ accumulation and suppress the WNK-SPAK/OSR1 pathway [54,62]. Effects of CaSR stimulation may further be mediated by alterations in intracellular levels of relevant second messengers such as Ca^2+^ or cAMP [63]. Endocrine stimulation of the TAL transport function critically depends on cAMP-generating hormones such as vasopressin (VP), PTH, calcitonin, or glucagon [64]. We have previously shown that VP recruits the WNK-SPAK/OSR1 pathway to facilitate the NKCC2 phosphorylation and function [49]. CaSR-induced PLC activation and release of Ca^2+^ from intracellular stores attenuate the hormone-dependent intracellular cAMP accumulation, which provides a potential explanation for the CaSR-dependent NKCC2 inhibition [63,65]. Rise in [Ca^2+^]_i_ in response to CaSR activation may exert further effects on WNKs. Binding of Ca^2+^ to the highly conserved locus of mammalian WNK kinases termed “acidic motif” has been proposed to directly modulate their activity [66]. To further exam this hypothesis, we have screened mammalian WNK isoforms for presence of canonical Ca^2+^-binding domains such as EF hand but failed to identify them either in the “acidic motif” or in the whole WNK sequences. Nevertheless, the “acidic motif” may bind cations due to enrichment in negatively-charged acidic residues [66]. This domain appears to mediate the interaction of WNK isoforms with KLHL3, which recruits the kinases to the E3 ubiquitin ligase complex for degradation [67,68]. It is unclear, whether binding of calcium ions within the “acidic motif” of WNK kinases modulates their interaction with KLHL3, but this possibility may explain the reported Ca^2+^-dependent stimulation of WNK4 kinase activity [66]. It has been speculated that gain-of-function FHHt-causing mutations of the “acidic motif” mimic the Ca^2+^-binding, thereby preventing degradation of WNKs and providing them with constitutive activity [66] (Figure 3). However, the physiological sense of Ca^2+^-dependent WNK stimulation in TAL remains obscure. According to this effect, CaSR activation in hypercalcemic conditions would facilitate the NKCC2 function, thereby increasing the driving force for paracellular reabsorption of divalent cations and aggravating hypercalcemia. An alternative mechanism of Ca^2+^-dependent WNK4 regulation has been proposed based on an interaction between WNK4 and CaM [69]. Screening of human WNK4 identified two potential CaM-binding motifs located within the amino acid sequences 492–552 and 1163–1212 [69]. A physical interaction of CaM with the WNK4 amino acid sequence 1163–1212 in the presence of Ca^2+^ has been proven using site-directed mutagenesis, binding assays, and functional studies in *Xenopus laevis* oocytes [69]. The obtained results suggested that binding of Ca^2+^/CaM to WNK4 inhibits its kinase activity via modulation of WNK4 phosphorylation by the serum and glucocorticoid-regulated kinase 1 [69]. The respective functional experiments used NKCC2 as an effector of WNK4 catalytic activity, suggesting a physiological relevance of this regulatory mechanism for the salt reabsorption in TAL [69]. Our own screening failed to identify presence of the canonical CaM-binding IQ-motifs in any of mammalian WNK isoforms. However, our results showed that the reported potential CaM-binding site located within the 1175–1194 amino acid sequence of human WNK4 is conserved across the mammalian WNK isoforms [69] (Figure 3). In addition to the CaM-dependent WNK inhibition, rise in [Ca^2+^]_i_ in response to CaSR activation may facilitate dephosphorylation of KLHL3 by calcineurin, thereby promoting its interaction with WNKs and their degradation [41]. Calcineurin may suppress the transport function of TAL via direct dephosphorylation of SPAK/OSR1 or NKCC2 as well [39].

### 3.2. CaSR Inhibits the Paracellular Ca^2+^ and Mg^2+^ Reabsorption

Several lines of evidence suggest that CaSR-induced rise in [Ca^2+^]_i_ activates the calcineurin- nuclear factor of activated T-cells (NFAT) signaling to enhance the expression of CLDN14 via a microRNA-dependent pathway [10,11,70]. Kidney-specific CaSR deletion resulted in decreased CLDN14 expression and reduced ability of the kidney to excrete calcium [30]. CLDN14 reduces the paracellular permeability of cortical TAL for divalent cations by physical interaction with CLDN16 and disruption of functional CLDN16/19 heterodimers [70]. CLDN14 is negatively regulated by two microRNAs, miR-9 and miR-374, which induce the posttranscriptional CLDN14 mRNA decay [70]. These microRNAs are downregulated by high dietary Ca^2+^ content and upregulated upon dietary Ca^2+^ depletion, whereas CLDN14-expression undergoes reciprocal changes [70]. Both miR-9 and miR-374 underlie the transcriptional control by the calcineurin-NFAT signaling, which stresses the key role of calcineurin in mediating effects of CaSR activation in TAL [11]. The role of WNKs in the regulation of paracellular TAL permeability received only minor attention so far. There is some general evidence for WNK-induced increase of paracellular epithelial permeability for chloride with potential impact on renal salt handling and blood pressure [71,72,73]. Most studies were performed in cultured Madin-Darby canine kidney cells and do not adequately reflect the TAL biology but may be relevant for other nephron segments [71,72,73]. In TAL, the dominant route for chloride reabsorption is the transcellular NKCC2-mediated transport (for review, [20]). Effects of WNKs on claudins conveying the TAL tight junctions permeability to monovalent (CLDN10) or divalent cations (CLDN14, 16, and 19) remain to be determined. According to the current knowledge, effects of CaSR on paracellular TAL permeability to Ca^2+^ and Mg^2+^ are chiefly mediated by the calcineurin-NFAT-microRNA-CLDN14 signaling (for review, [74]).

## 4. CaSR Function in JGA

The juxtaglomerular apparatus (JGA) comprises MD cells, renin-producing JG cells of the afferent arteriole, and extraglomerular mesangial cells. CaSR-induced stimulation of COX-2 activity in MD cells is expected to promote renin biosynthesis via paracrine mechanisms [75]. This assumption received an indirect support by analysis of patients with autosomal dominant hypocalcemia and Bartter-like syndrome due to gain-of-function mutation in the CaSR gene [76]. The analyzed patients exhibited a hyperreninemia, which was corrected by a COX-inhibitor indomethacin [76]. In contrast, animal studies provided several lines of evidence for inhibitory effects of CaSR activation on renin secretion and plasma renin activity [77,78]. Since the animal experiments followed acute protocols of CaSR stimulation, their results may reflect direct effects of CaSR activation in JG cells [77,78]. Effects of CaSR activation on renin biosynthesis and release in JG cells are likely mediated by calcineurin, since inhibition of calcineurin in cultured JG cells promotes renin exocytosis [79,80].

## 5. CaSR Function in DCT

DCT has major impact on the urinary excretion of NaCl, K^+^, and divalent cations (for review, [81]). This nephron segment comprises the early (DCT1) and the late portions (DCT2) differing in their morphological and functional properties. DCT1 consists of only one cell population and performs the transcellular NaCl reabsorption exclusively via NCC at the apical side. In contrast, DCT2 contains scattered intercalated cells (ICs) and co-expresses NCC and ENaC in the apical membrane of its DCT2 cells [82,83,84]. DCT2 exhibits significant expression of Ca^2+^ and Mg^2+^ transport proteins mediating their transcellular reabsorption, whereas DCT1 lacks the most of these proteins [82,85,86]. The endocrine regulation of DCT1 vs. DCT2 differs in several aspects. DCT2 cells express the 11β-hydroxysteroid dehydrogenase 2 and are directly responsive to aldosterone [87]. In contrast, regulation of DCT1 function by aldosterone is likely indirect and may be mediated by the hormone-induced alterations of potassium homeostasis [23]. The transport activity of NCC defines the Na^+^ load of the downstream CNT and CD, thereby modulating the Na^+^-coupled K^+^ excretion in these segments (for review, [81]). NCC activity may further modulate the Ca^2+^ handling along the distal nephron, although the underlying mechanisms are still debatable [88]. It has been argued that NCC-dependent modulation of intracellular Na^+^ [Na^+^]_i_ content may reciprocally affect the basolateral Na^+^-coupled Ca^2+^ efflux via the sodium-calcium exchanger 1 (NCX1) in DCT2 cells (for review, [89]). This mechanism may provide an explanation for the thiazide-induced hypocalciuria [88]. Alternatively, suppression of NCC activity by thiazide and the associated NaCl wasting may lead to compensatory stimulation of NaCl and Ca^2+^ reabsorption in the proximal tubule [90].

In contrast to TAL, where CaSR signaling inhibits the NaCl reabsorption, activation of CaSR in DCT cells has been suggested to stimulate NCC in order to compensate for NaCl loss in the preceding TAL [13]. CaSR-induced NCC activation is mediated by WNK4, at least in cell culture and *Xenopus laevis* oocytes transfected with the two products [13]. In these ex vivo systems, CaSR activation has been shown to promote PKC-dependent phosphorylation of WNK4 and KLHL3 resulting in enhanced WNK4 kinase activity and reduced degradation of the kinase via the KLHL3-dependent pathway [13,91]. Notably, WNK4 is expressed both in cortical TAL and DCT, where it mediates activation of NKCC2 and NCC, respectively [92,93]. In this context, the opposite effects of CaSR activation on NKCC2 vs. NCC deserve further clarification. Several C-terminally-truncated WNK4 variants have been identified in the kidney, including variants with increased, as well as decreased catalytic activity towards SPAK [94]. The described C-terminal truncations may eliminate the putative CaM-binding site (amino acids 1175–1194), thereby abrogating the inhibitory effects of [Ca^2+^]_i_ on WNK4 activity [69,94]. Assuming distinct distribution of WNK4-variants between TAL and DCT, it is tempting to speculate that the truncated WNK4 variants lacking the inhibitory CaM-binding site but preserving the SPAK-binding motif are enriched in DCT. The CaSR-induced rise in [Ca^2+^]_i_ in DCT would then activate truncated WNK4 variants via Ca^2+^-binding to their acidic domain without competitive inhibitory effects mediated by C-terminal interaction with CaM [66,69,94]. Future studies using isolated rodent nephron segments are mandatory for resolving this issue. WNK4 activity can be potentiated by an interaction with the truncated, kidney-specific WNK1 variant (KS-WNK1), which abrogates the inhibitory effects of [Cl^−^]_i_ [95,96,97]. In view of the fact that KS-WNK1 expression is much higher in DCT compared to the other nephron segment, this mechanism may play a role in distinct effects of CaSR-activation between TAL and DCT as well [95,98,99]. It is also worth mentioning that CaSR exerts inhibitory effects on Kir4.1, which may result from physical or functional interactions between the receptors [55,56]. Kir4.1 is critical to the WNK4-SPAK activity and resulting activating NCC phosphorylation, especially in hypokalemic conditions [96,100,101]. From this perspective, basolateral CaSR activation may suppress the WNK-SPAK-NCC signaling due to Kir4.1 inhibition. Since the results on physical and functional interactions between CaSR and Kir4.1 were obtained ex vivo in *Xenopus laevis* oocytes and human embryonic kidney (HEK293) cells [55,56], further studies are warranted to clarify whether CaSR interferes with the Kir4.1 activity in vivo as well. CaSR may reside both in the apical and basolateral membranes of DCT cells and effects of plasma- vs. urinary Ca^2+^ on NCC may be different, i.e., inhibition by hypercalcemia vs. stimulation in hypercalciuric conditions [5,13,56,89].

DCT2 and the ensuing CNT are capable of transcellular Ca^2+^ reabsorption, which is an active process mediated by the transient receptor potential vanilloid member 5 (TRPV5) at the luminal side, calbindin D28K as intracellular carrier, as well as the basolateral NCX1 and plasma membrane Ca^2+^-ATPase [86]. CaSR activation has been shown to stimulate the TRPV5 activity by inducing its activating, PKC-dependent phosphorylation [9]. WNK4 has been implicated in the TRPV5 regulation, but the data remains controversial [102,103,104,105,106]. While some studies suggested that WNK4 may enhance the surface expression and activity of TRPV5 [102,106], other studies reported the opposite results [104]. Effects of WNK4 on TRPV5 may be mediated by non-catalytic interactions of the kinase with proteins of the vesicular trafficking machinery such as syntaxins or caveolins [103,104,106]. Controversial effects of WNK4 on the TRPV5 surface expression may be related with co-factors modulating the TRPV5 membrane stability such as the Na^+^/H^+^ exchanger regulating factor 2 (NHERF2) [106]. Experiments in *Xenopus laevis* oocytes showed that WNK4 may promote both, the exocytotic TRPV5 trafficking to increase its surface expression, as well as the channel internalization [105,106]. Co-expression of NHERF2 stabilized TRPV5 in the plasma membrane, thus preventing its internalization and switching WNK4 to the TRPV5-activating mode [106]. The inhibitory effects of WNK4 depend on caveolae-mediated TRPV5 internalization, as shown in cultured HEK293 cells [104]. Along the same line, caveolae-mediated endocytosis has been implicated in the modulation of TRPV5 surface expression by uromodulin in HEK293 cells [107]. Evaluation of caveolin-1 and caveolae distribution in the mouse and rat kidneys showed their basolateral presence in DCT2 and principal cells (PCs) of CNT/CD but failed to confirm the apical localization of caveolae in vivo [108,109]. Apical caveolin-1 trafficking can be induced by supraphysiological doses of a vasopressin V2 receptor agonist desmopressin, as has been shown in vasopressin-deficient Brattleboro rats [110]. Nevertheless, no apical formation of caveolae was observed under this condition as well [110]. Therefore, the role of caveolae-mediated endocytosis in the regulation of TRPV5 surface expression in vivo warrants further study. Interestingly, co-expression of TRPV5 with NCC in *Xenopus laevis* oocytes exerted an inhibitory effect on the TRPV5 activity in the presence of WNK4 [105]. The NCC-dependent TRPV5 inhibition was potentiated by FHHt-causing WNK4 mutants suggesting that NCC activity may interfere with the functional TRPV5 at the cellular level, as has been stated previously [88,105]. Collectively, earlier studies provide several lines of evidence for regulation of TRPV5 and NCC by the CaSR-WNK4 pathway and suggest that effects of this signaling pathway may be modulated by various co-factors such as NHERF2, PKC, or further molecular switches to be identified.

## 6. CaSR Function in CNT and CD

CNT and CD consist of PCs and ICs. PCs perform transcellular reabsorption of water via the luminal aquaporin 2 (AQP2) and basolateral AQP3 and AQP4. Na^+^ reabsorption is mediated by ENaC and electrogenically coupled to K^+^ secretion via ROMK in this cell type (for review [25]). In addition, PCs of CNT express Ca^2+^ and Mg^2+^ transport proteins mediating the transcellular reabsorption of divalent cations [85,86]. ICs fulfil important tasks in the renal acid-base handling and comprise the proton-secreting type A cells (A-ICs), bicarbonate-secreting type B cells (B-ICs), as well as an intermediate cell population likely reflecting a transition state between the two cell types (non-A/non-B-ICs). Apart from the acid-base transport, B-ICs mediate transcellular Cl^−^ reabsorption via the Cl^−^/HCO_3_^−^ exchanger pendrin, whereas A-ICs contribute to K^+^ excretion via the H^+^/K^+^-ATPase (for review [111]). Localization studies suggested heterogeneous CaSR expression in distinct subsets of PCs and ICs, although precise characterization of CaSR-expressing IC types and fractions, as well as intracellular receptor distribution in different CNT/CD cell types remain to be clarified [5,46,48].

The role of CaSR in CNT received only minor attention but studies on regulation of TRPV5 and other Ca^2+^-transporting proteins permit an assumption that CaSR activation may promote Ca^2+^-reabsorption in CNT in analogy to DCT2 [9]. Stimulation of CaSR in isolated CDs by increasing extracellular Ca^2+^ levels has been shown to promote H^+^ secretion, which may result in urinary acidification in vivo [112]. This effect was due to CaSR-induced activation of the vacuolar H^+^-ATPase (V-ATPase) residing in the apical membrane of A-ICs [112]. The resulting urinary acidification is critical for prevention of nephrolithiasis in conditions of enhanced luminal Ca^2+^ delivery [112]. Interestingly, stimulation of vasopressin V1a receptor in A-ICs has been shown to exert similar effects mediated by rise in [Ca^2+^]_i_ and activation of V-ATPase [113]. Apart from the urinary acidification, CaSR-dependent suppression of AQP2 may help to prevent urinary stones due to decreased water reabsorption and dilution of urinary Ca^2+^ [112]. Available data on expression and function of WNK isoforms in ICs is scarce, whereas PCs exhibit significant expression of WNK4 and may express other WNK variants as well [92]. Interactions between the full-length WNK1 (L-WNK1), KS-WNK1, and WNK4 in PCs affect ENaC and ROMK functions [114,115]. Little information is available on protein networks and potential paracrine interactions mediating effects of CaSR activation in distinct CD cell types. However, several CaSR gene polymorphisms have been linked to kidney stones in human, suggesting that intact CaSR function in CD is critical to renal adaptations to enhanced luminal Ca^2+^ concentrations (for review, [116]).

## 7. Translational Perspectives

**Figure 4:**
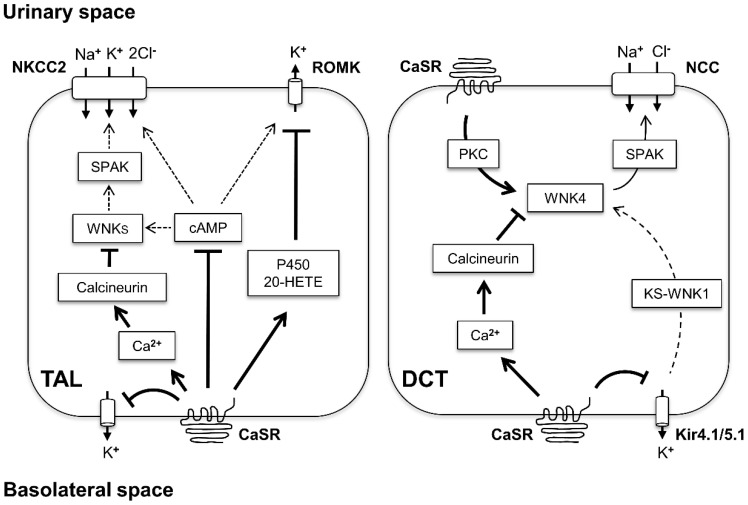
Schematic drawing showing effects of calcium-sensing receptor (CaSR) activation on the with-no-lysine [K] kinases (WNKs) signaling in the thick ascending limb (TAL; left side of the panel) and distal convoluted tubule (DCT; right side of the panel) cells. In TAL, CaSR resides in the basolateral membrane. Stimulation of the receptor leads to enhanced production of P450-derived prostanoids, which inhibit the rat outer medullary potassium channel (ROMK). CaSR-induced Ca^2+^ release from intracellular stores activates calcineurin, thereby suppressing the WNK-SPAK/OSR1 kinase signaling. Similarly, CaSR-induced reduction of intracellular cAMP levels may contribute to WNK-SPAK/OSR1 inactivation as well. The net effect of CaSR activation is the inhibition of WNK-SPAK/OSR1 signaling and suppression of NKCC2 phosphorylation and function. In DCT, CaSR may reside both in the luminal and basolateral membranes. The luminal CaSR activates the WNK4-SPAK pathway by stimulating PKC and inducing the activating phosphorylation of WNK4. In contrast, the basolateral CaSR may suppress WNK4 via stimulation of calcineurin or by interfering with Kir4.1/5.1-mediated potassium sensing and preventing KS-WNK1 from facilitating WNK4 activity. The net effects of CaSR-activation on the WNK-SPAK activity in DCT cells may depend on various modulating factors. T-shaped lines indicate inhibitory CaSR effects, arrows show stimulating CaSR effects, and dashed arrows reflect attenuation of WNK-dependent NKCC or NCC stimulation; the thickness of the arrows reflects strength of effects.

The primary function of CaSR in the kidney is to adjust the urinary Ca^2+^ excretion to the needs of the body. To achieve this purpose, CaSR signaling regulates a wide range of electrolyte transporters and channels, which are directly or indirectly involved in the Ca^2+^ reabsorption along the nephron and collecting duct system. With respect to salt-handling, global effects of CaSR hyperactivity due to gain-of-function mutations in the human CaSR gene recapitulate a Bartter-like syndrome with moderate salt wasting but rather severe hypocalcemia compared to the classical Bartter syndrome variants [28,76]. Missense mutations in human CaSR gene cause familial hypocalciuric hypercalcemia (FHH) or neonatal severe hyperparathyroidism (NSHPT) dependent on the allele dose [117,118]. Heterozygous and homozygous CaSR-knockout mice exhibit similar gene dose-dependent phenotypes reflecting human FHH or NSHPT, respectively [119]. Effects of CaSR inactivation on sodium balance and blood pressure have not been extensively characterized [30]. CaSR agonists (calcimimetics) or antagonists (calcilytics) may be helpful for management of Ca^2+^ metabolism in endocrine disorders (for review, [120]). Calcimimetics may provide clinical benefits in patients with chronic kidney disease or recurrent kidney stones as well (for review, [120,121]). However, application of calcimimetics or calcilytics for corrections of the renal salt handling may be associated with substantial hypo- or hypercalciuria and cardiovascular complications [121]. Selective targeting of CaSR in distinct segments of nephron or collecting duct system may provide options for the fine-tuning of sodium balance but requires development of the respective drug carrier systems. Alternatively, further elucidation of protein networks mediating effects of CaSR activation in TAL, DCT, CNT, and CD may identify candidates for pharmacological targeting, permitting a selective stimulation or inhibition of CaSR signaling in these segments. In this context, WNK isoforms are emerging as molecular links enabling coordination of calcium and sodium homeostasis. WNK-mediated effects of CaSR activation relevant to sodium reabsorption along the distal nephron are schematized in Figure 4. Advanced characterization of renal CaSR signaling and resulting functional interactions between segments of the distal nephron and collecting duct system bears the potential for improved management of electrolyte and blood pressure disorders.
